# Characterizing Diagnostic Delays in Metachromatic Leukodystrophy: A Real‐World Data Approach

**DOI:** 10.1002/jimd.70049

**Published:** 2025-06-02

**Authors:** Ali Mohajer, Anjana Sevagamoorthy, Karen Bean, Sylvia Mutua, Francis Pang, Laura Ann Adang

**Affiliations:** ^1^ Qral Group New Jersey New Jersey USA; ^2^ Division of Neurology Children's Hospital of Philadelphia Philadelphia Pennsylvania USA; ^3^ Orchard Therapeutics London UK; ^4^ Department of Neurology, Perelman School of Medicine University of Pennsylvania Philadelphia Pennsylvania USA

**Keywords:** diagnostic barriers, diagnostic delay, metachromatic leukodystrophy, prodromal features, real world data

## Abstract

Neurodegeneration in metachromatic leukodystrophy (MLD) may be preceded by systemic complications. Characterization of these features is critical to define barriers to early diagnosis and treatment eligibility for gene therapy. We utilized medical billing (claims) datasets and a natural history study to capture pre‐diagnosis MLD‐related events. MLD‐related events (ICD‐10‐CM codes) were aggregated into system‐based diagnosis clusters, and time to MLD diagnosis (TTD) computed for each organ‐system diagnosis cluster. Differences in TTD distribution, instantaneous diagnosis hazard, and survival to MLD diagnosis were compared by sex and payor type. TTD and regression from onset of first symptoms were described using median and inter‐quartile range. The claims dataset identified 174 MLD cases (diagnosis ≤ 6 years old) with 14 diagnosed within the first year of life. General neurologic concerns (*n* = 138; median 257 days pre‐diagnosis), gastrointestinal (*n* = 137; 231 days), seizures (*n* = 48; 236 days), ophthalmologic (*n* = 46; 362 days), and language‐related events (*n* = 41; 267 days) were common. Time to MLD diagnosis from onset of prodromal clusters was longer for children with non‐commercial insurance: most prominent with seizures (survival logrank *p* value < 0.02) and non‐degenerative neurological symptoms (survival logrank *p* value < 0.04). Similar findings were noted in our analysis of a second claims dataset. The natural history cohort demonstrated a similar pattern of prodromal disease features and delayed diagnosis. This study defines barriers to MLD diagnosis and highlights prodromal periods of pre‐regression symptomatology, further supporting the need for early screening in this fatal disorder of childhood.

## 1 Introduction

Metachromatic leukodystrophy (MLD) is a rare [1] neurodegenerative disorder due to low arylsulfatase A activity (Arsa, encoded by *ARSA*). MLD is associated with progressive central and peripheral demyelination and death [2, 3, 4, 5, 6]. The subtypes of MLD are defined by the age of disease onset: late infantile (LI; onset before 2.5 years), early juvenile (EJ; 2.5–7 years), late juvenile (LJ;7–16 years), and adult. The LI and EJ are the most common subtypes and are collectively referred to as “early onset” MLD [7]. Ex vivo gene therapy (atidarsagene autotemcel) has demonstrated benefit in presymptomatic (LI and EJ) or early symptomatic (EJ only) cases [8, 9, 10, 11, 12, 13, 14, 15, 16]. As such, there is a limited window for potential intervention and a critical need for early diagnosis.

The neurodegeneration of MLD is well characterized and results in the progressive loss of mobility and communication skills [4, 8, 14, 17]. Children affected by this inborn error of metabolism may also experience early developmental delay, feeding concerns, gallbladder disease, and eye movement abnormalities [7, 18, 19, 20, 21, 22, 23, 24, 25]. Ophthalmologic concerns in particular, including strabismus (reported in 17 toddlers with late onset MLD) and nystagmus, have been reported to precede neurologic symptoms [25, 26, 27]. Because of its rarity and non‐specific presentation, a prolonged period before diagnosis is common [28]. As such, at diagnosis, most individuals are not eligible for gene therapy [28].

The need for earlier diagnosis has driven pilot newborn screening programs, which measure residual ARSA activity, sulfatide levels, in combination with *ARSA* genotyping [29, 30, 31]. In tandem with newborn screening, it is critical to define the prodromal period of disease so that cases can be diagnosed in the prodromal period when children may still be eligible for intervention.

The International Classification of Diseases (ICD), maintained by the World Health Organization (WHO), is the global standard for coding diagnoses, symptoms, and procedures [32, 33, 34]. Used by providers, auditors, and researchers, ICD codes (ICD‐10‐CM) support documentation, billing, and epidemiological research. Based on ICD coding, medical claims databases (payor‐system databases) are an alternative type of electronic record collection that support collation of patient‐level de‐identified information, irrespective of the hospital or care provider. ICD‐10‐CM codes, tracked through claims databases, can be used to map high‐level information related to the medical journey across hospital systems.

In this study, we evaluate the early features of disease prior to diagnosis using a payor‐system database, specifically focusing on differences by sex and payor type (private vs. public insurance) in the time from onset of symptoms to diagnosis, and compare this to a natural history study which used medical records to retrospectively map medical events prior to diagnosis [28].

## 2 Materials and Methods

### 2.1 Standard Protocol Approvals, Registrations, and Patient Consents

We performed a retrospective cohort study using a natural history study and claims databases. The natural history study cohort patients were consented under the Myelin Disorders Biorepository Project (MDBP) (IRB #14‐011236) as part of the Global Leukodystrophy Initiative Clinical Trial Network (GLIA‐CTN). Written informed consent for research was obtained from all participants or their legally authorized representatives if unable to provide consent due to age or diminished capacity. Records in claims datasets are de‐identified; therefore, the analysis is not classified as human subjects research.

### 2.2 Claims Database Cohort

**FIGURE 1:**
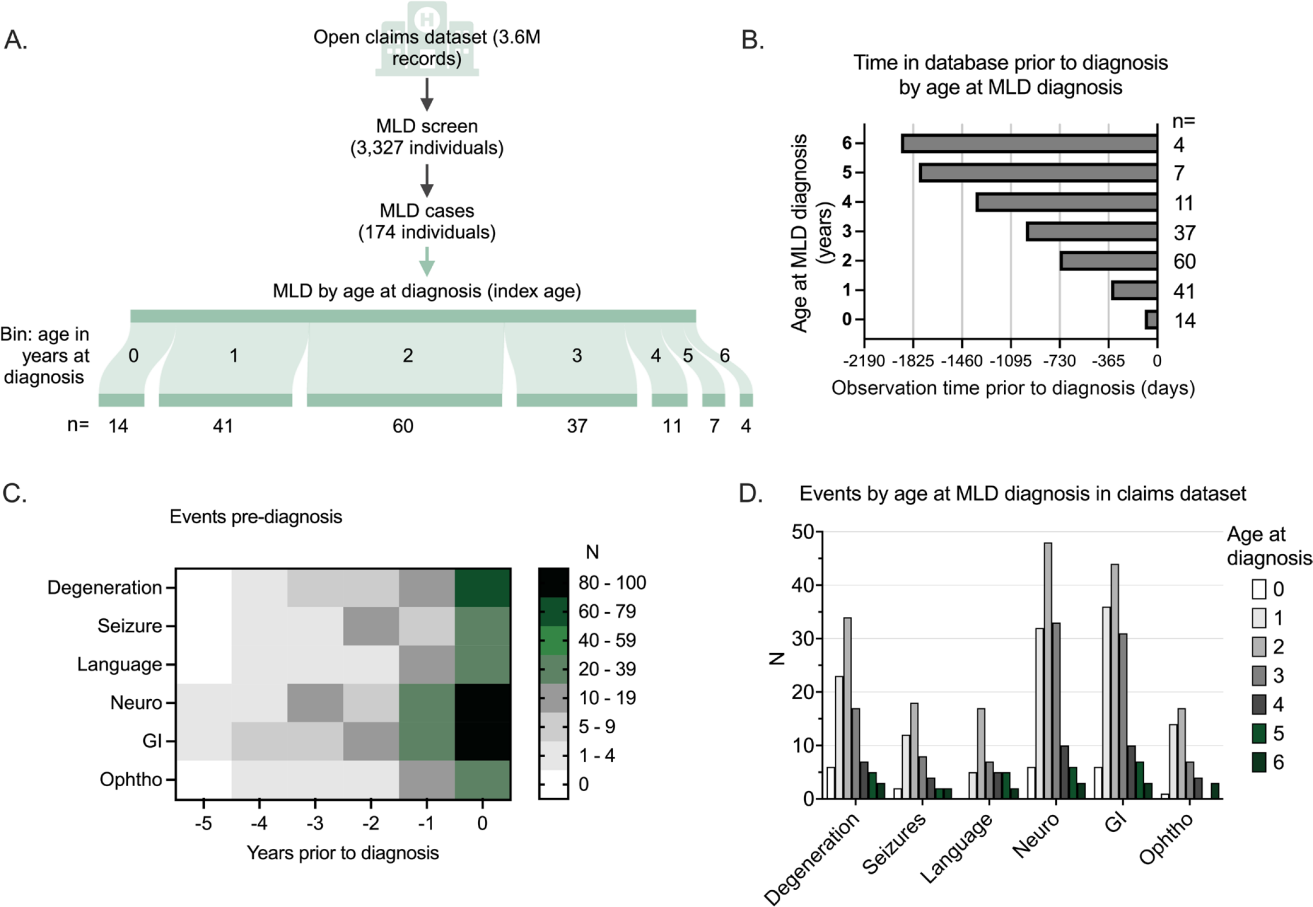
MLD cohort description. (A) In total, 174 cases of MLD were identified within the claims database with a diagnosis of MLD before 7 years. This dataset includes more than 3.6 million individuals. This figure was created in Biorender. (B) Duration of pre‐MLD observation time by age at MLD diagnosis (range 0–6 years old). (C, D) All events coded in the medical system prior to the diagnosis of MLD were captured. Events were clustered into clinical features: Seizures (*n* = 48), language/cognitive concerns (*n* = 41), neurologic features (*n* = 138), gastrointestinal or feeding concerns (*n* = 137), ophthalmologic (*n* = 46), and suspicion of neurologic disease (*n* = 95). (C) A heatmap represents the non‐cumulative events occurring within each category in the years prior to diagnosis. (D) Event occurrence was stratified by age at diagnosis of MLD (range: 0–6 years old).

The claims dataset included 3327 patients with at least one use of the MLD‐specific International Classification of Disease, 10th Revision, Clinical Modification (ICD‐10‐CM) E75.25 diagnosis code (Table 1, Figure 1A). A primary cohort of 174 patients was selected from Veeva Compass Patient, an anonymized, longitudinal open US‐based medical claims dataset with procedures and diagnoses at patient‐level granularity. This data source comprises over 300 million patients, with 31.9 million born on or after Jan 1, 2017. Data availability spans from January 1, 2017, to Feb 28, 2024. Patients were only eligible for study inclusion if they had a year of birth of 2017 or later and at least two distinct medical claims with an E75.25 diagnosis separated by at least 30 days. Data are detailed at the patient/claim/line‐item/day level and include payor type (e.g., commercial, Medicaid, Medicare). Available demographics include sex and year of birth, and patients are geographically well‐dispersed (Figure S1). The median time between an eligible subject's first appearance in the claim's dataset and their index MLD diagnosis was 658 days (IQR: 334.25–952.25, Table 1).

**TABLE 1:** Primary claims cohort characteristics.

Year of birth	Individuals (*N*)		Sex (*N*)			Payor type (*N*)		Median MLD index age	Median observation days before MLD diagnosis (IQR)
	Universe	Eligible	F	M	UK	Comm	Public		
2023	3 600 137	1		1		1		0	62
2022	4 141 138	3	1	2		2	1	1 (1–1)	314 (280–337.5)
2021	4 294 461	21	8	13		16	5	2 (1–2)	342 (222–586)
2020	4 630 728	25	12	12	1	18	7	2 (1–2)	573 (223–812)
2019	4 557 586	31	14	17		23	8	2 (1.5–2.5)	818 (469–985)
2018	4 730 820	34	24	10		23	11	2 (1.25–3)	703 (360–916.75)
2017	5 134 483	59	28	31		45	14	3 (2–4)	837 (522–1330)

*Note:* A total of 174 subjects were identified from 2017 to 2023 within the Veeva claims database.

Abbreviations: Comm, commercial; F, female; M, male; UK, unknown; Universe, primary claims dataset universe.

Medical events are recorded at the subject/day level, so sequences of events were mapped for granular longitudinal analysis. Subject‐level birth year is available across the span of the datasets, so approximate subject age for each event was also computed.

In the Veeva Compass Patient dataset, payor information was detailed at the line‐item level, enabling the assignment of primary coverage types (e.g., commercial, public) for the primary cohort. Also, as the ICD‐10‐CM codes are linked to dates in the Veeva database, they allow comparison between events and thereby assessment of multiple encounters in the medical system prior to diagnosis.

A complementary MLD cohort (*n* = 248 patients) was identified within the Symphony Health PatientSource, a second anonymized, longitudinal open US‐based medical claims dataset with patient‐level granularity (Supporting Information). The results of this dataset are presented in Figure S2. Payor type data were not available from the Symphony dataset.

### 2.3 Diagnosis Determination in the Claims Dataset

In the primary dataset (Veeva), confirmed MLD cases were defined by the use of ICD‐10‐CM E75.25 on two distinct claim IDs separated by at least 30 days in the primary cohort, and by the use of E75.25 on a date following an initial use of either ICD‐9‐CM 330.3 (“Cerebral degeneration of childhood in other diseases classified elsewhere”) or ICD‐10‐CM E75.25 (“Metachromatic Leukodystrophy”) in the secondary cohort. For confirmed patients, the age at MLD diagnosis, referred to as the index year of life, was defined as the year of the first use of E75.25 minus the year of birth. The age at the occurrence of other diagnoses was determined similarly. When diagnoses are grouped or clustered, the first occurrence of any claim with a diagnosis in the grouping is used.

### 2.4 Symptom Cluster Determination in the Claims Dataset

Each medical claim is linked with specific ICD‐10‐CM codes representing concerns addressed during the medical encounter. There are often multiple similar codes representing similar clinical complaints. To minimize bias from differences in ICD‐10‐CM code selection, we grouped potential early complications of MLD by related ICD‐10‐CM codes (Table 2) [4, 7, 19, 25, 35, 36]. This allowed harmonization across the medical encounters irrespective of the specific billing code. Systemic clusters were ophthalmologic and gastrointestinal (GI)/feeding related. The GI/feeding related symptoms category included dysphagia, gall bladder abnormalities, and failure to thrive. The neurologic clusters comprised codes related to language/cognitive concerns, seizures, neurologic features unrelated to language or cognition, and suspicion of neurologic disease (Table 2). All neurologic concerns such as tone, developmental delay, movement abnormalities, and psychomotor regression were included within the neurologic features category. The suspicion of neurologic disease category included codes indicative of the first suspicion of neurologic disease origin, such as co‐occurring codes related to other neurodegenerative conditions as well as general codes for white matter abnormalities. Since each code was binned into at most one category, clusters are non‐overlapping. Within a cluster, the earliest occurrence of a code is used for patient‐level longitudinal analysis. For example, if two different seizure‐related codes were used longitudinally for the same individual, then only the first event within the category was used to define the age at seizure onset.

**TABLE 2:** Clusters of clinical features were created to minimize differences in coding practices and duplicate counting of subjects within a category.

	Description	Code	Patient count (all)
Ophthalmology	Unspecified optic atrophy	H4720	1
	Other optic atrophy, bilateral	H47293	2
	Unspecified strabismus	H509	7
	Other specified disorders of binocular movement	H518	4
	Unspecified disorder of binocular movement	H519	3
	Unspecified visual loss	H547	8
	Unspecified nystagmus	H5500	24
	Congenital nystagmus	H5501	16
	Other forms of nystagmus	H5509	13
	Other irregular eye movements	H5589	2
GI	Calculus of gallbladder with acute cholecystitis without obstruction	K8000	1
	Calculus of gallbladder without cholecystitis without obstruction	K8020	1
	Acute cholecystitis	K810	1
	Cholesterolosis of gallbladder	K824	1
	Other specified diseases of gallbladder	K828	1
	Other specified diseases of biliary tract	K838	1
	Dysphagia, unspecified	R1310	49
	Dysphagia, oral phase	R1311	10
	Dysphagia, oropharyngeal phase	R1312	33
	Other dysphagia	R1319	14
	Failure to thrive (child)	R6251	62
	Other lack of expected normal physiological development in childhood	R6259	12
	Feeding difficulties	R633	59
	Unspecified lack of expected normal physiological development in childhood	R6250	93
Neurologic features	Specific developmental disorder of motor function	F82	59
	Difficulty in walking, not elsewhere classified	R262	9
	Other abnormalities of gait and mobility	R2689	24
	Unspecified abnormalities of gait and mobility	R269	29
	Ataxia, unspecified	R270	11
	Other lack of coordination	R278	9
	Unspecified lack of coordination	R279	7
	Psychological and behavioral factors associated with disorders or diseases classified elsewhere	F54	1
	Pervasive developmental disorder, unspecified	F849	1
	Other disorders of psychological development	F88	53
	Unspecified disorder of psychological development	F89	7
	Stereotyped movement disorders	F984	4
	Extrapyramidal and movement disorder, unspecified	G259	5
	Spastic quadriplegic cerebral palsy	G800	10

	Spastic diplegic cerebral palsy	G801	8
	Cerebral palsy, unspecified	G809	11
	Quadriplegia, unspecified	G8250	3
	Abnormal head movements	R250	1
	Other abnormal involuntary movements	R258	13
	Unspecified abnormal involuntary movements	R259	15
	Abnormal reflex	R292	4
	Abnormal posture	R293	6
	Other symptoms and signs involving the musculoskeletal system	R29898	38
	Other symptoms and signs involving appearance and behavior	R4689	5
	Dysarthria and anarthria	R471	1
	Weakness	R531	16
	Delayed milestone in childhood	R620	56
	Idiopathic progressive neuropathy	G603	1
	Guillain‐Barre syndrome	G610	1
	Chronic inflammatory demyelinating polyneuritis	G6181	3
	Polyneuropathy, unspecified	G629	4
	Dystonia, unspecified	G249	17
	Contracture of muscle, multiple sites	M6249	2
	Muscle weakness (generalized)	M6281	35
	Other muscle spasm	M62838	8
	Other specified disorders of muscle	M6289	48
	Disorder of muscle, unspecified	M629	10
	Congenital hypotonia	P942	25
	Cramp and spasm	R252	17
	Altered mental status, unspecified	R4182	23
Irritability and anger	R454	7
Suspicion of neurologic disease	Carnitine deficiency due to inborn errors of metabolism	E7142	11
	X‐linked adrenoleukodystrophy, unspecified type	E71529	12
	Disorder of amino‐acid metabolism, unspecified	E729	12
	Other disorders of galactose metabolism	E7429	11
	Other sphingolipidosis	E7529	68
	Hereditary spastic paraplegia	G114	1
	Other specified degenerative diseases of nervous system	G3189	7
	Degenerative disease of nervous system, unspecified	G319	15
	Other abnormal findings on diagnostic imaging of central nervous system	R9089	30
	Abnormal brain scan	R9402	2
	White matter disease, unspecified	R9082	21
Language/cognition	Mild cognitive impairment of uncertain or unknown etiology	G3184	1
	Mixed receptive‐expressive language disorder	F802	14
	Speech and language development delay due to hearing loss	F804	5
	Other developmental disorders of speech and language	F8089	7
	Developmental disorder of speech and language, unspecified	F809	24
	Other speech disturbances	R4789	7
	Unspecified speech disturbances	R479	1
Seizure	Localization‐related (focal) (partial) idiopathic epilepsy and epileptic syndromes with seizures of localized onset, not intractable, without status epilepticus	G40009	2
	Localization‐related (focal) (partial) symptomatic epilepsy and epileptic syndromes with simple partial seizures, not intractable, with status epilepticus	G40101	2
	Localization‐related (focal) (partial) symptomatic epilepsy and epileptic syndromes with simple partial seizures, not intractable, without status epilepticus	G40109	4
	Localization‐related (focal) (partial) symptomatic epilepsy and epileptic syndromes with simple partial seizures, intractable, without status epilepticus	G40119	2
	Localization‐related (focal) (partial) symptomatic epilepsy and epileptic syndromes with complex partial seizures, not intractable, with status epilepticus	G40201	1
	Localization‐related (focal) (partial) symptomatic epilepsy and epileptic syndromes with complex partial seizures, not intractable, without status epilepticus	G40209	3
	Localization‐related (focal) (partial) symptomatic epilepsy and epileptic syndromes with complex partial seizures, intractable, with status epilepticus	G40211	1
	Localization‐related (focal) (partial) symptomatic epilepsy and epileptic syndromes with complex partial seizures, intractable, without status epilepticus	G40219	2
	Other generalized epilepsy and epileptic syndromes, not intractable, without status epilepticus	G40409	11
	Other seizures	G4089	10
	Unspecified convulsions	R569	43

### 2.5 Payor‐Type Designation Within the Claims Dataset

In the primary cohort, patient‐level primary payor type was assigned as “Commercial” if the majority of line items in the pre‐index period were designated as “Commercial” by the data provider; otherwise, it was assigned as “Public/Other.” For example, a patient with 45% “Commercial,” 35% “Medicaid,” and 20% “Cash” line items in the period preceding their first ICD‐10‐CM E75.25 diagnosis would be designated as “Commercial”.

### 2.6 Natural History Cohort

The natural history study criteria included an international cohort of subjects with confirmed late infantile or early juvenile MLD by molecular and/or biochemical confirmation and consistent medical history. Only children with symptomatic disease prior to diagnosis were included. In contrast to the claims dataset, the natural history cohort was not restricted by birth year and included all individuals meeting the eligibility criteria. Medical records were reviewed prior to diagnosis and key medical events along with age at time of occurrence of the event were collected, as previously described [28]. Data collection was limited to events occurring prior to diagnosis. The identified medical events were categorized as neurologic concerns, psychomotor regression, gastrointestinal or feeding concerns, and ophthalmologic abnormalities. Neurologic concerns included features related to tone, developmental delay, seizures, and psychomotor impairments unrelated to regression. Psychomotor regression included events related to loss of previously acquired skills. Medical records and the extracted variables were collected and stored in a secure Research Electronic Data Capture (REDCap) database. REDCap is a web application designed for securely storing clinical and translational research data [37, 38]. Ages were rounded to the nearest 2 weeks.

Three children were excluded from the analysis because of early diagnosis leading to treatment or missing symptomatic medical records (drop out). As such, all children from the natural history cohort had neurologic symptoms prior to diagnosis.

### 2.7 Disease Onset Determination

As per consensus guidelines, the onset of MLD was defined as the age at the first neurologic symptom [39]. If the initial disease‐related event was non‐neurologic, the age at the first systemic symptom was used to define the prodromal period. This study was limited to children with an MLD diagnosis at or prior to 6 years (claims database) or symptom onset prior to 6 years (natural history cohort).

### 2.8 Statistical Approach

Sex was considered a biologic variable and was used as a variable for analyses (Table S2). Statistical analysis of claims data was performed using R version 4.3.3 with updated libraries (e.g., survival_3.5–8). Where the normality of underlying distributions could not be assumed, nonparametric tests were used (e.g., Mann–Whitney *U*). For survival analysis stratified according to payor type, the “Commercial” type was used as the referent, and for stratification according to sex, “Female” was used as the referent. Linear regression of log‐scaled Schoenfeld residuals against time was used to check for violation of assumptions necessary for hazard modeling. Time granularity is at the calendar day level, except where otherwise noted. The number of natural history subjects within each systemic and neurologic symptom cluster was described using counts and proportions. Median and interquartile range were used to describe time from onset of each symptom category, including psychomotor regression, to MLD diagnosis and time from first symptom to psychomotor regression. Longitudinal disease progression at the subject level was illustrated using a swimmer lane plot. PRISM graphical software was used to generate descriptive statistics and create graphs for the natural history cohort (Prism GraphPad 9.2.0 [34]).

## 3 Results

### 3.1 Claims Cohort

In total, 174 cases were included in the MLD cohort (Table 1, Figure 1A). The birth year ranged from 2017 to 2023, and this cohort included 87 females, 86 males, and 1 sex unknown. These subjects had a median of 658 days (IQR: 334.25–952.25) between first recorded observation in claims data and index MLD diagnosis (Table 1, Figure 1B). The majority of cases were diagnosed prior to 2.5 years of age, with 66.1% of the cohort being diagnosed before age 3 (*n* = 115).

Events noted in the claims database prior to diagnosis were clustered into key neurologic categories (Figure 1C,D; Table 2): language/cognitive concerns (*n* = 41), seizures (*n* = 48), neurologic features (*n* = 138), and suspicion of neurologic disease (*n* = 95). The “neurologic features” category, which included tone, delayed milestones and gait disturbances, occurred a median of 256.5 days (IQR: 90.25–502.25) prior to diagnosis. This “neurologic features” category included several common medical codes, including “Delayed milestone in childhood” (R620; *n* = 56) and “Specific developmental disorder of motor function” (F82; *n* = 59 cases).

Systemic complaints were also common within the pre‐diagnosis MLD population, including gastrointestinal or feeding (*n* = 137) and ophthalmologic concerns (*n* = 46) (Table 3, Figure S1). The common codes within the gastrointestinal or feeding concerns cluster included failure to thrive (R6251, *n* = 62) and feeding difficulties (R633, *n* = 59). Gallbladder disease, a known complication of MLD, was only noted prior to diagnosis in two cases.

**TABLE 3:** The time to diagnosis was compared by early clinical feature and type of insurance within the claims database.

Cluster	Measure											
	Count (*N*)					Median TTD (IQR)			*p*			
	Total	Com	Public	F	M	All	Com	Public	Cox	KM	Schoen	W
Neuro	138	107	31	69	69	256.5 (90.25–502.25)	232 (87.5–438.5)	314 (92.5–796)	0.0412	0.0398	0.4760	0.1606
Language/Cognitive concerns	41	30	11	21	20	267 (100–572)	297.5 (101.25–565.5)	251 (96.5–547)	0.9811	0.9723	0.7393	0.8598
Seizures	48	34	14	23	25	235.5 (83.25–746.75)	157.5 (45.75–582.75)	626.5 (214–951.75)	0.0153	0.0128	0.6174	0.0152
GI/Feeding‐related	137	102	35	66	71	231 (82–572)	202 (77.75–453.75)	339 (122–729)	0.1825	0.1848	0.4709	0.1297
Ophtho	46	33	13	19	27	362 (63.5–574.5)	168 (63–464)	526 (258–804)	0.2175	0.2143	0.5664	0.1757
Suspicion of neurologic disease	95	76	19	42	53	143 (40–444.5)	120.5 (33–401)	273 (132.5–541.5)	0.1361	0.1336	0.3731	0.0740

*Note:* Multiple statistical approaches were used to compare by payor type, including Cox Proportional Hazards (logrank; *p* < 0.05: Significant difference in the instantaneous risk of E75.25 diagnosis); Kaplan Meier (KM) survival (logrank; *p* < 0.05: Significant difference in the time‐dependent probability of acquiring an E75.25 diagnosis); Schoen: Test on time‐dependence of scaled Schoenfeld residuals (*p* > 0.05: No significant evidence of violation of assumption regarding constant proportional hazard over time), and Wilcoxon (W) rank sum (aka Mann–Whitney *U*) test (*p* < 0.05): Significant difference in distribution of time to E75.25 diagnosis (non‐parametric test to compare medians).

Abbreviations: Com, Commercial; F, female; GI, gastrointestinal; KM, Kaplan Meier; M, male; neuro, neurologic features; ophtho, ophthalmologic concerns; TTD, time to MLD diagnosis (days).

There was an even distribution by sex in the cohort (female, *n* = 87/174). No differences in the median time to diagnosis within the symptom clusters by sex were noted (Figure S3, Table S1). As such, the sexes were combined for subsequent analyses.

In the primary claims cohort, events of systemic and neurologic involvement up to 1849 days preceding diagnosis were observed in a large proportion of the sample (92.5%; *n* = 161). To evaluate if these results were replicable across platforms, we utilized a complementary claims cohort to evaluate whether these patterns were consistent (Figure S1, Table S2). Similar to the primary claims cohort, cases of MLD often had a preceding period of neurologic and systemic symptoms prior to diagnosis.

**FIGURE 2:**
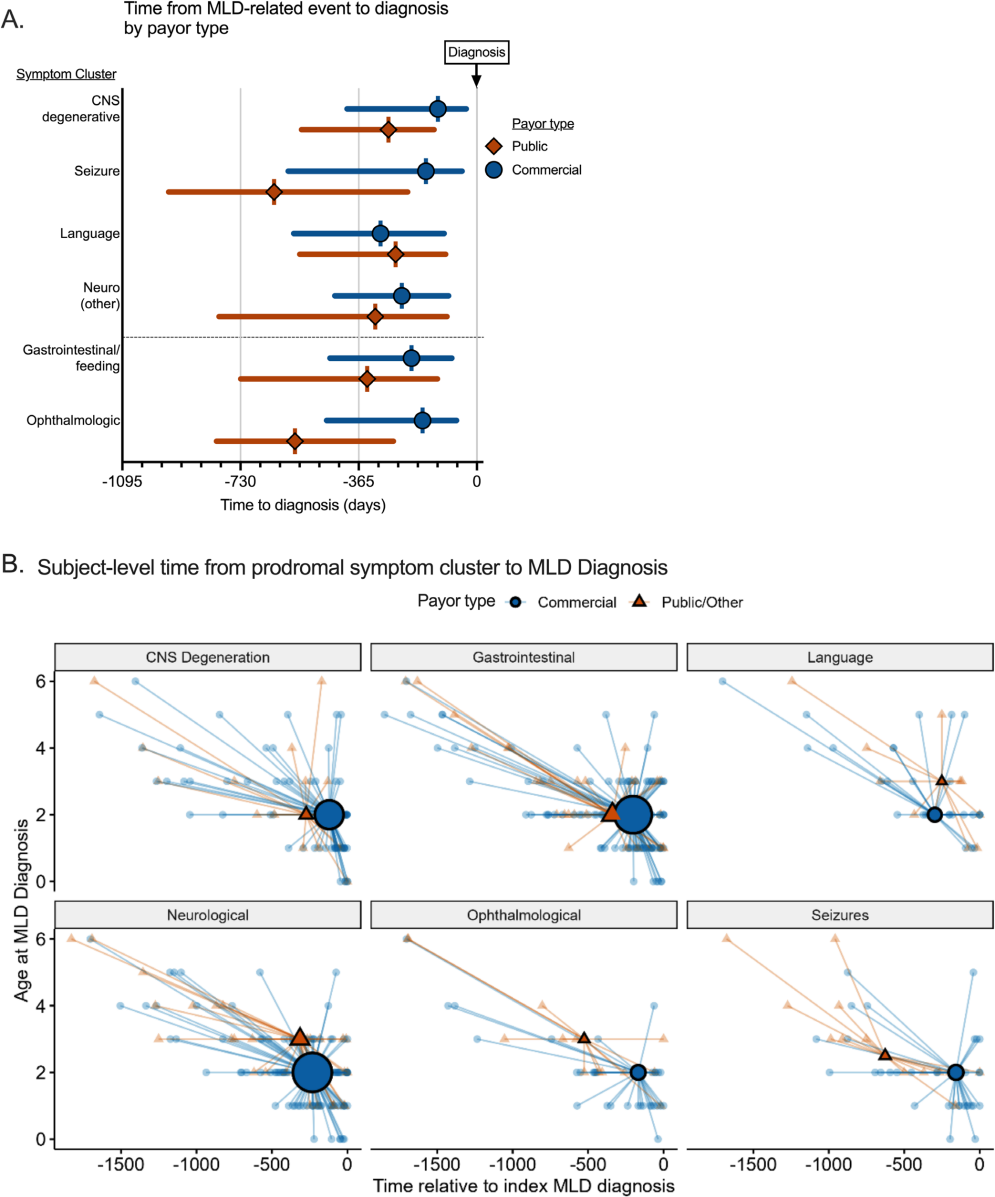
Signs and symptoms prior to diagnosis in MLD by medical coding within a payor database. All events coded in the medical system prior to the diagnosis of MLD were captured. Events were clustered into clinical features: Neurologic features (*n* = 138), seizures (*n* = 48), language/cognitive concerns (*n* = 41), gastrointestinal or feeding concerns (*n* = 137), ophthalmologic (*n* = 46), and suspicion of neurologic disease (*n* = 95). (A) The median time to diagnosis is shown by the symbol with the interquartile range shown in the error bars. Diagnosis is shown as the line at time 0 on the *x*‐axis. A dotted *y*‐axis line separates neurologic from systemic features. Subjects with events within each category were divided by public (dark diamond) versus private payor (light circle). (B) The temporal relationship between features of disease and diagnosis at the subject level stratified by insurance. All events coded in the medical system prior to the diagnosis of MLD were captured and were clustered into CNS degeneration, gastrointestinal, language/cognitive concerns, neurologic features, ophthalmologic, and seizures. The large central icon indicated the median age in years at MLD diagnosis (*y*‐axis) versus the temporal proximity of the event to MLD diagnosis (*x*‐axis). Each small symbol represents the time‐based relationship between the preceding medical event and the diagnosis of MLD. Symbols positioned to the right represent that the event occurred proximal to diagnosis, and symbols positioned low indicate that diagnosis occurred early in life. The size of the population with each feature cluster is shown by the size of the symbol.

**FIGURE 3:**
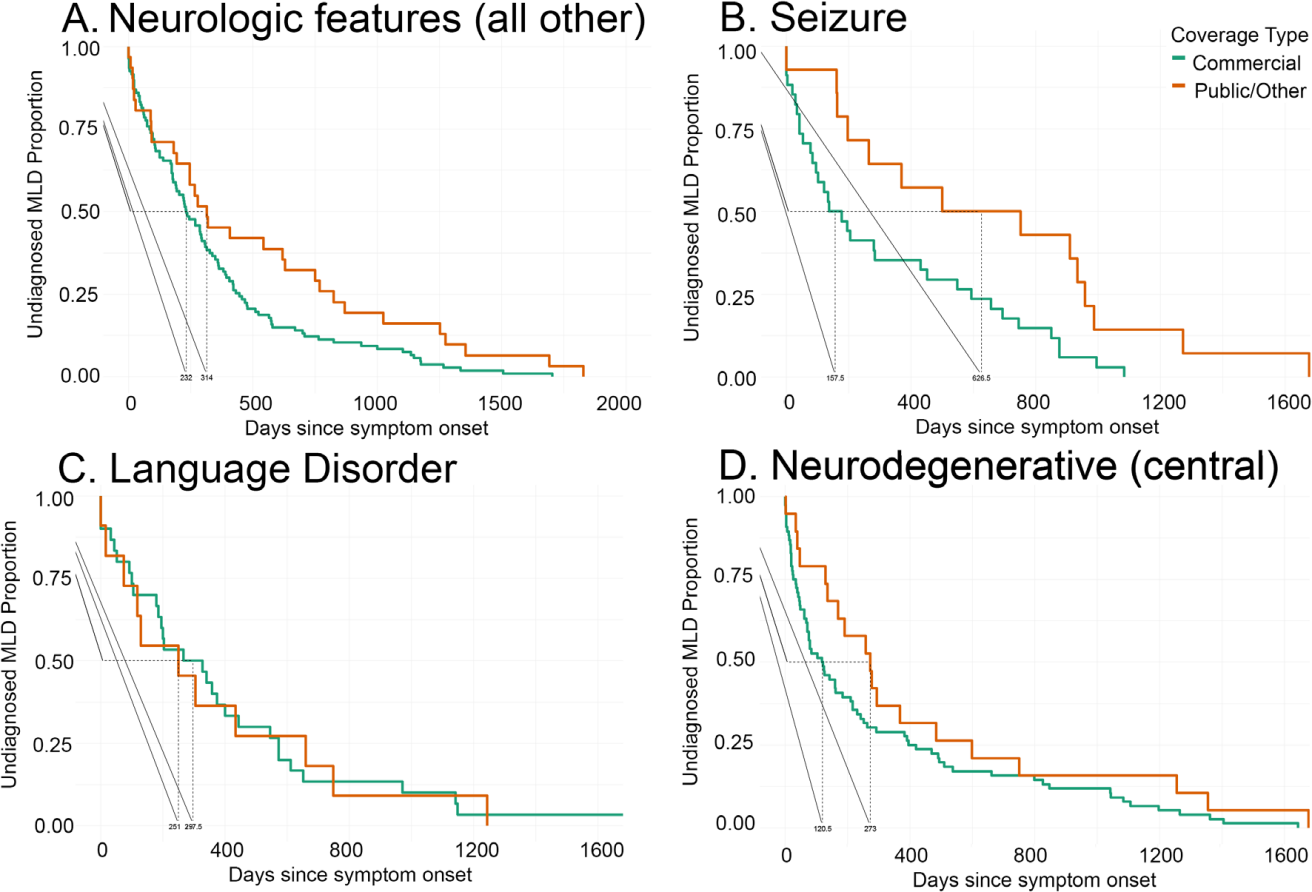
The time from neurologic prediagnostic event to diagnosis was captured in the MLD cohort. The time from neurologic features (*n* = 138; A), seizures (*n* = 48; B), language/cognition (*n* = 41, C), and suspicion of neurologic disease (*n* = 95, D) to MLD diagnosis is presented by Kaplan–Meier curve. The medical codes included in each category are mutually exclusive. The “neurologic features” category includes developmental delay, tone, and gross motor abnormalities. Events were compared by payor status: Public (orange color) vs. private (green color) (see Table 3 for statistical comparisons).

**FIGURE 4:**
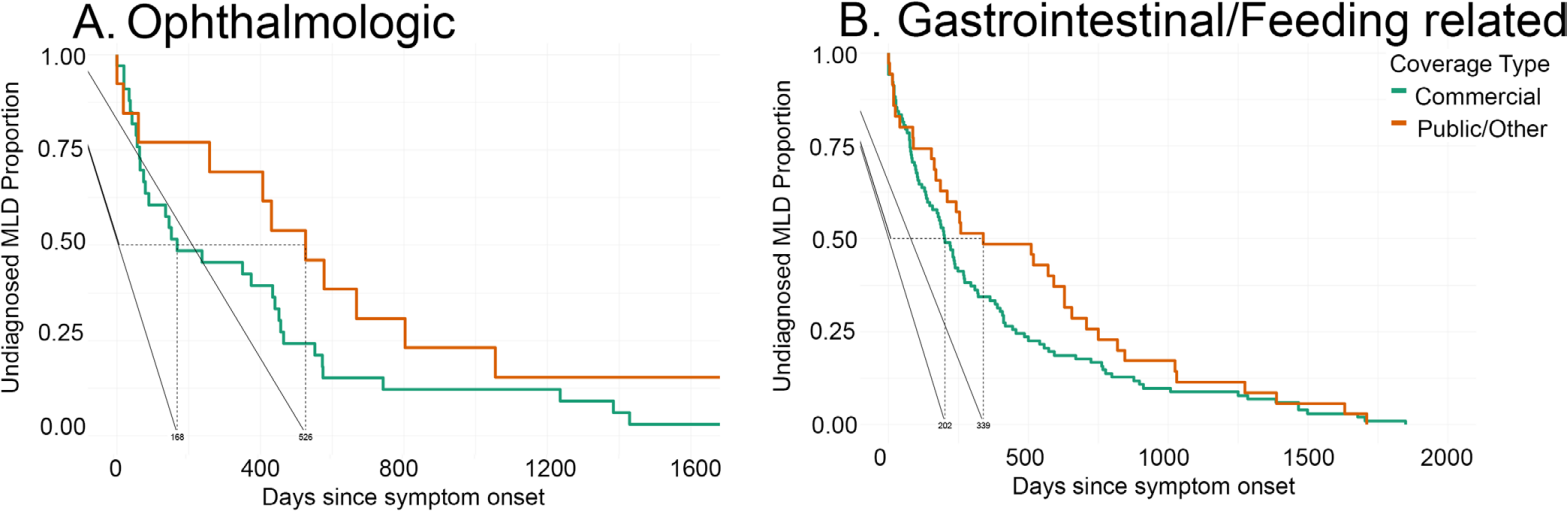
The time from systemic prodromal event to diagnosis was captured in the MLD cohort. The time from systemic feature ophthalmologic (A; *n* = 46) and gastrointestinal or feeding‐related (B; *n* = 137) to MLD diagnosis is presented by Kaplan–Meier curve. The medical codes included in each category are mutually exclusive. Payor status was compared: Public (orange) vs. private (green). Events were compared by payor status: Public (orange color) vs. private (green color) (see Table 3 for statistical comparisons).

In both payor type cohorts, there was a prodrome of systemic and neurologic concerns prior to diagnosis. Next, we sought to evaluate if payor type impacted how children engaged with the medical system (Table 3, Figures 2, 3, 4). At a line‐item level prior to MLD diagnosis, the payor type in the primary claims cohort was 67% commercial and median time‐in‐data prior to MLD diagnosis was 1.8 years. Children with commercial coverage had shorter median time to diagnosis across 5 of the 6 studied disease feature clusters (for statistical comparisons see Table 2). Among children in whom prodromal symptoms are detected, those with commercial coverage also reached diagnosis at a younger age overall (MDN 2 versus 3) and in 4 of the 6 studied symptom clusters. This temporal relationship between features of disease and diagnosis at the subject level stratified by insurance is illustrated in Figure 2B, which comprises a large central icon indicating the median age at MLD diagnosis with icon size reflective of population size and small symbols representing the time‐based relationship between the preceding medical event and the diagnosis of MLD. Symbols positioned to the right represent that the event occurred proximal to diagnosis, and symbols positioned low indicate that diagnosis occurred early in life.

### 3.2 Natural History Cohort

**FIGURE 5:**
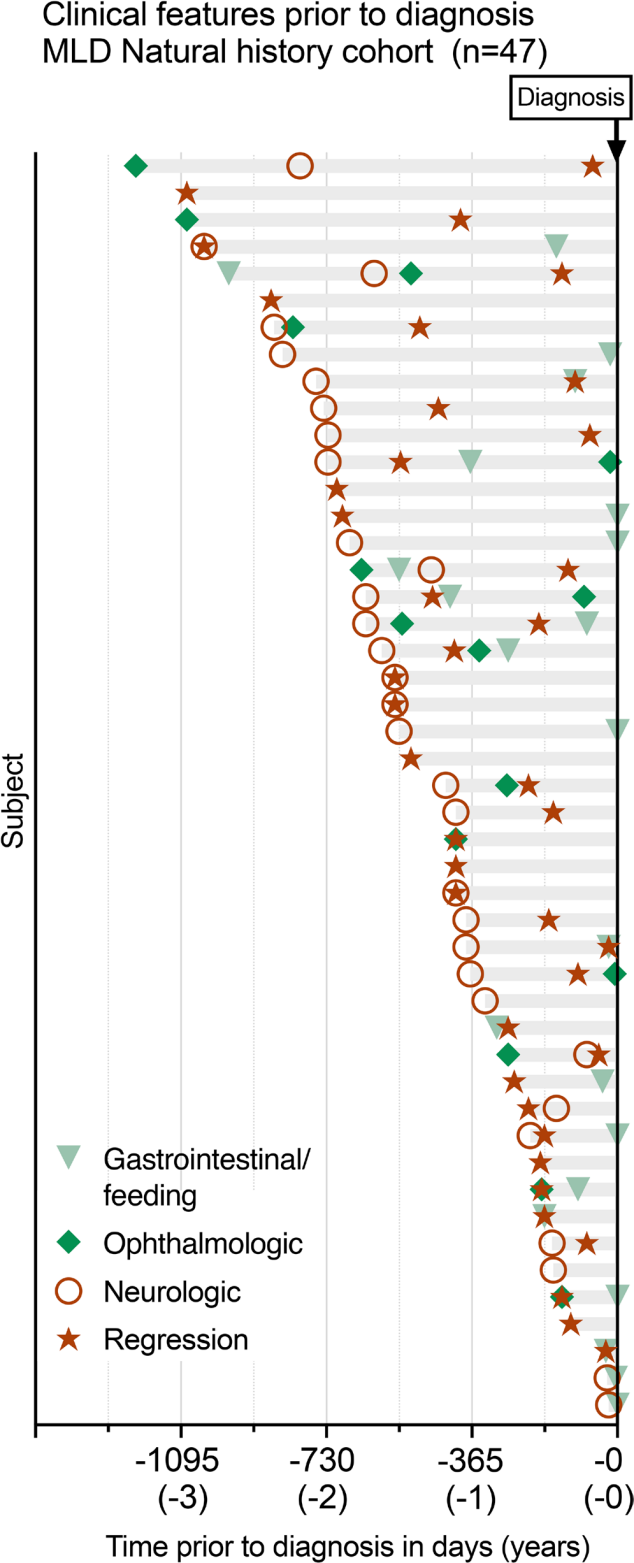
Individual disease course from the natural history study shows features prior to diagnosis in MLD. The medical records of 47 subjects with MLD were reviewed to identify early features of disease, which were categorized as related to gastrointestinal or feeding (light triangle), ophthalmologic (dark triangle), neurologic concerns (unrelated to psychomotor regression; open circle), and psychomotor regression (star). Regression was defined as loss of previously acquired developmental milestones. The age at diagnosis is shown at time 0 on the *x*‐axis. Each horizontal bar represents an individual.

Finally, we sought to capture the early symptomatology of MLD through an alternative approach using an existing retrospective natural history study. In total, 47 symptomatic children affected by early‐onset MLD were identified (LI *n* = 38, Table 4). Subject‐level datapoints on the longitudinal progression of disease with time of onset of each symptom category from diagnosis are illustrated in Figure 5.

**TABLE 4:** Description of natural history cohort.

	Age at first event in the medical records (years)				Age at MLD diagnosis (years)
	Neurologic concerns	Psychomotor regression	Ophthalmologic concerns	Gastrointestinal or feeding related	
*N*	31	40	15	22	47
Median	1.3	1.8	1.7	2.1	2.6
IQR	1.0, 1.5	1.4, 2.4	1.4, 2.5	1.4, 2.9	2.2, 3.8
Mean	1.9	2.3	1.9	2.4	3.2
Std. deviation	1.7	1.5	0.8	1.6	1.6
Time from event to diagnosis (years)					
*N*	31	40	15	22	
Median	1.5	0.6	1.0	0.2	
IQR	2.0, 0.9	1.4, 0.3	1.8, −0.4	0.8, 0.0	
Mean	1.3	0.9	1.2	0.5	
Std. deviation	0.7	0.7	1.0	0.7	

*Note:* In total, 47 individuals with disease onset prior to diagnosis were identified in the retrospective natural history study. The medical records were reviewed to identify early features of disease, which were categorized as neurologic concerns, psychomotor regression (e.g., loss of previously acquired skills), ophthalmologic, or related to gastrointestinal or feeding.

All children included in this cohort had neurologic symptoms prior to diagnosis, including 32 (68%) children who had notable neurologic concerns prior to regression. These early neurologic features were documented a median of 548 days prior to diagnosis (IQR: −726, −332). This included 25 children with noted gross motor concerns prior to diagnosis, often failure to gain ambulation, and 8 children with tone abnormalities (e.g., dystonia, spasticity, and hypotonia). Seizures were noted in 5 children, and language concerns were noted in 4 children. Regression prior to diagnosis was also common. Across the cohort, regression occurred a median of 223 days prior to diagnosis (IQR: −488, −128, *n* = 40).

Systemic concerns prior to diagnosis were noted in 29 of the 47 children (62%). Gastrointestinal concerns (*n* = 22) were primarily failure to thrive or dysphagia, and these events were noted a median of 57 days prior to diagnosis (IQR: −231, −0). Ophthalmologic abnormalities (*n* = 15) were primarily strabismus or nystagmus and were noted a median of 223 days prior to diagnosis (IQR: −642, −139). Within the natural history cohort, extra‐neurologic symptoms (GI and ophthalmologic categories) were noted prior to the first neurologic symptom in 6/29 patients. Overall, extra‐neurologic symptoms occurred in the first 12 months of life in 2/29 patients.

## 4 Discussion

The availability of time‐sensitive therapies presents novel challenges and opportunities in delivering appropriate healthcare for the MLD population. Understanding the health trajectory of the disease is a critical piece of defining how the medical system can meet the needs of the MLD community. The goal is to identify all children who would benefit from treatment within the window of clinical eligibility for treatment. As such, the optimal system should focus on early diagnosis and close monitoring [7, 39]. Additionally, even post‐implementation of newborn screening, there will be cases where disease subtype will not be able to be pre‐symptomatically determined [2, 40, 41, 42, 43, 44, 45, 46]. Likely each individual will have a unique pattern of prodromal symptoms, including developmental delay, feeding‐related issues such as dysphagia, and gall bladder abnormalities, and thus the team responsible for identifying treatment‐eligible individuals must be adaptable and responsive. A deeper understanding of the prodromal disease features can inform earlier subtype prediction and thus therapy eligibility.

Using real‐world data, both through claims databases and a medical records‐based retrospective natural history study [28], we were able to identify a subset of children with early feeding and GI symptoms prior to the onset of neurologic symptoms or a diagnosis of early‐onset MLD. For example, failure to thrive and feeding difficulties were commonly noted prior to diagnosis. Eye movement abnormalities were also noted, including 32 patients with nystagmus. Recognition of these early features has the potential to identify children at risk for imminent neurologic decline during the window where they are still eligible for intervention.

Even once children had onset of neurologic symptoms of disease, there was often a prolonged period before diagnosis during which children were labeled by isolated features of MLD, including delayed developmental milestones or “anger and irritability”. This delay in diagnostic is also evident from the Veeva Compass Patient dataset, which includes patients who were diagnosed in or before the year 2023.

Recent international guidelines recommend atidarsagene autotemcel, an FDA approved ex vivo gene therapy, within the first year of life for presymptomatic patients with late‐infantile‐ and early juvenile‐ forms of MLD [7, 39]. Of note, only 8% of children (14 of 174) were diagnosed within the recommended window for treatment eligibility, the first year of life [7]. It is not possible to elucidate through the database if the early diagnosis was because of older affected siblings, although this is likely as this was described in original clinical trials where a large proportion of treated children were only identified because they were younger siblings of an affected older child [47]. The ratio of private: public payor in this early diagnosis sub‐cohort was similar to the payor distribution in the overall MLD population.

The remaining cohort majority was diagnosed after a year of age, which is likely outside the window of potential intervention. The gap from neurologic onset to diagnosis was longer in children with public insurance, most notable when seizures were noted. This critical difference is likely multifactorial, including likelihood to seek care, access to healthcare, as well as institutional and diagnostic biases related to ability to access advanced diagnostics and providers with disease‐specific expertise. Regardless, the implications are serious and underscore the importance of early screening.

For this study, we characterized the health trajectory of an MLD cohort using real‐world data. As opposed to electronic health records‐based (EHR) research which generally fails to capture care across institutions and platforms, claims databases capture all encounters at the subject level. Our approach, however, has important limitations. As the study data are based on the US healthcare system, the findings related to payors and time to diagnosis may not be generalizable in particular to countries exclusively using a public insurance system. However, the evidence suggests existing diagnostic delays across countries and payor systems [45]. Similarly, within our cohort, we found significant delay in diagnosis across the cohort regardless of payor system.

All retrospective approaches are influenced by biases in documentation and care. Both ethnicity and race are known to influence the determination of the severity of medical concerns, which would be indirectly reflected in the medical documentation and medical coding [48, 49]. The public payor status and use of claims databases are more likely to include a representative population compared to the natural history cohort [50]. An additional limitation is that the claims databases rely on ICD‐10‐CM codes without access to medical records to allow for confirmation. As such, the data available are dependent on how the provider coded medical concerns. This affects both the diagnosis of MLD as well as disease‐specific symptoms. In particular, we are unable to verify the MLD diagnosis by standard methods (genotype, enzyme level, or sulfatides) or confirm standard definitions of disease‐related symptoms. In addition, the timepoint available for the onset of a given prodromal symptom represents the first use of the ICD‐10‐CM code within the electronic health record and may be delayed compared to when the family first had concern. For example, a child may have had strabismus for several months prior to medical evaluation or diagnosis based on access to care. If this is the case, then our study could potentially be underestimating the time from the onset of prodromal symptoms to MLD diagnosis. To this end, our cohort only captured patients diagnosed in the final year of the database (2023). Based on the range of eligible individuals within the prior years and the incidence of MLD, it is probable that approximately 20–30 patients will have been ultimately diagnosed with MLD.

To overcome challenges related to the use of the MLD code as the anchor for MLD diagnosis in this study, we limited the data set to individuals for whom the MLD code was used longitudinally. These more stringent criteria decreased the number of identified cases from 3327 to 174, which is consistent with the predicted incidence of disease [7, 24, 25, 36, 39, 51, 52]. It is also possible that this process also included those for whom MLD was later excluded, such as those with the ultra‐rare MLD‐related disorders multiple sulfatase deficiency or PSAP deficiency [53]. Second, in our cohort identification, we restricted the claims cohort to subjects born on or after Jan 1st, 2017, to prevent left censoring of any pre‐MLD diagnosis events and allow for full claims history from birth. Changes to clinical practice, including diagnostics, over the years may limit the comparability between cohorts. Therefore, we describe the findings from the claims and the natural history cohorts to complement each other. Of note, the findings from both the two claims cohorts and the natural history cohort support the overall findings of delayed diagnosis and a pre‐regression prodromal period. Finally, claims data is independent from confirmation documentation. For this reason, in the claims dataset, psychomotor regression was grouped within the broad category of neurologic features. This limits the study of temporality between the psychomotor regression‐related medical events and other prodromal symptoms using the claims dataset alone. We addressed this limitation through the inclusion of granular natural history data with documentation of the onset of regression.

Overall, this study underscores that early signs and symptoms of MLD begin before disease regression and families are presenting to medical care with symptoms months to years prior to diagnosis. This study also underscores the critical need for early screening platforms and close monitoring of ambiguous cases, both because MLD begins earlier than previously defined and because, without appropriate support, the medical system fails to identify the children within the window for intervention. Lastly, there were differences in the timing to diagnosis based on insurance payor, which has the potential to impact clinical treatment eligibility.

## Author Contributions

L.A.A. and K.B. conceptualized the study. K.B. and F.P. contributed the claims dataset and critically appraised the study design. A.S. and S.M. extracted the natural history data from medical records. A.M. performed the analysis. All authors contributed to the interpretation of the study data. A.M., A.S., and L.A.A. contributed to the generation of figures and table for the study. L.A.A., A.M., and A.S. wrote the final manuscript. All authors reviewed, edited, and corrected the manuscript. L.A.A. is finally responsible and the guarantor.

## Ethics Statement

Subjects are enrolled and data is collected upon obtaining the written informed consent.

## Consent

The study is performed as part of the Myelin Disease Biorepository Project (MDBP) that is approved by the Children's Hospital of Philadelphia (CHOP) Institutional Review Board (IRB approval number: 14‐011236).

## Conflicts of Interest

Ali Mohajer received funding from Orchard Therapeutics. Karen Bean and Francis Pang are employees of Orchard Therapeutics. Laura Ann Adang is a consultant and receives research support from Takeda, Orchard, and Biogen. Laura Ann Adang is on the Scientific Advisory board for CureMLD and the MLD Foundation. Anjana Sevagamoorthy and Sylvia Mutua have no disclosures to report.

## Supporting information


**Data S1.** Supporting Information.

## Data Availability

The payor databases used in this study are publicly available resources. The data that support the findings of the study are not publicly available due to privacy and ethical concerns.
