# Supplementary material for: Characterizing Diagnostic Delays in Metachromatic Leukodystrophy: A Real‐World Data Approach
Source: J Inherit Metab Dis. 2025 Jun 2;48(4):e70049. doi: 10.1002/jimd.70049 (PMC12130617; doi:10.1002/jimd.70049)
Supplement: Supplementary file 1 — Data S1. Supporting Information. [file JIMD-48-0-s001.docx]

**Supplemental materials.**

**Complementary claims database cohort**

A complementary cohort of 248 patients was identified within a second anonymized, longitudinal open US-based medical claims dataset that includes dispensed prescriptions, procedures, and diagnoses at patient-level granularity (Symphony Health PatientSource^®^). This dataset included the date of claim, with available demographics including sex and year of birth, but omitted the payer information available in Veeva. Records from each patient’s first year of life are masked to year-of-birth granularity. Data availability spans from July 1, 2013, to August 31, 2022. Events were recorded at a year-of-life granularity in the first year, transitioning to daily granularity thereafter.

**Supplemental Figure 1. Geographic distribution in Veeva database.**

**
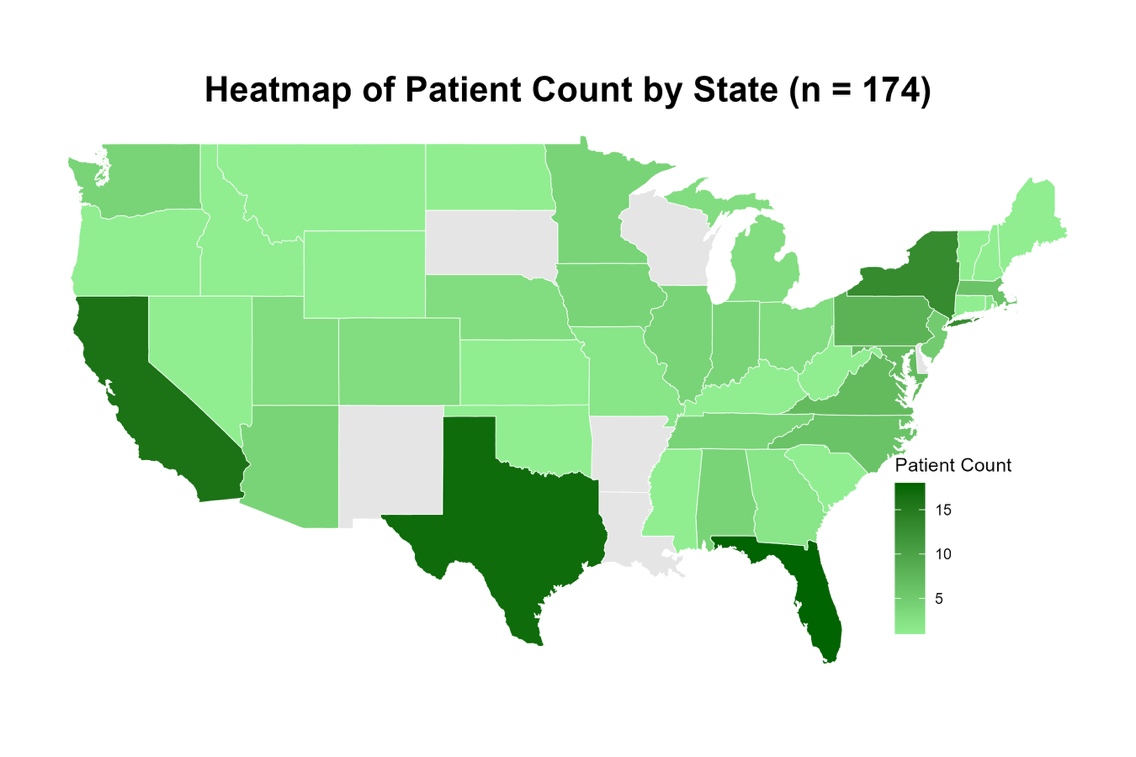
**

**Supplemental Figure 2.** Heatmaps of key events related to MLD diagnosis from the Symphony claims dataset, listed in order of frequency within the cohort.

I.

| A. Unspecific lack of expected normal physiological development in childhood (R62.50) | B. Feeding difficulties (R63.3) |
| --- | --- |
| 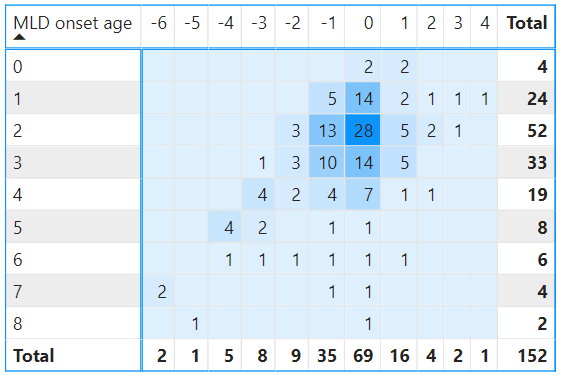 | 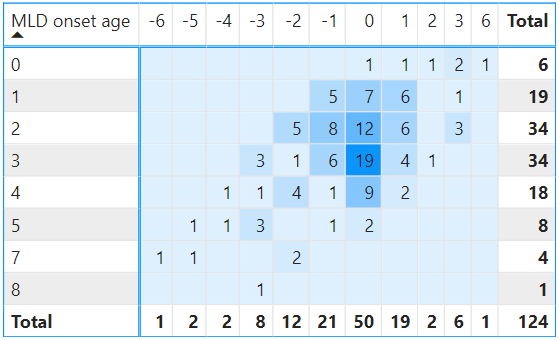 |
| C. Dysphagia, unspecific (R13.10) | D. Failure to thrive (R62.51) |
| 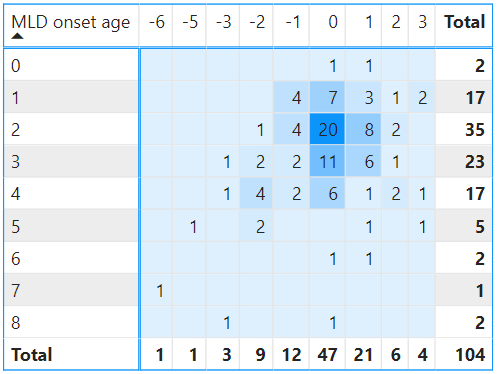 | 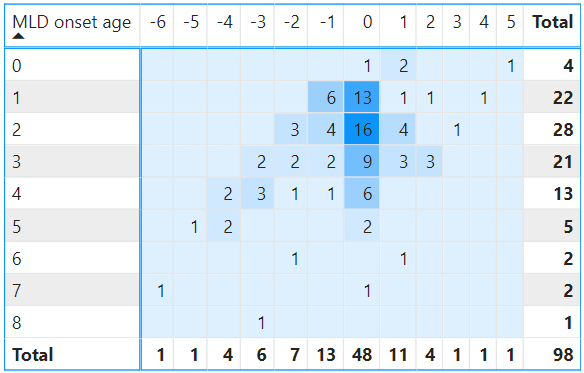 |
| E. Other disorders of psychological development (F88) | F. Delayed developmental milestone in childhood (R62.0) |
| 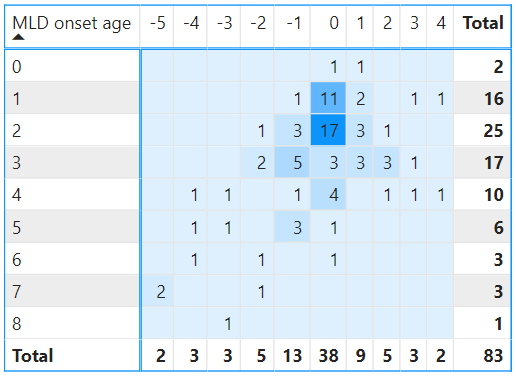 | 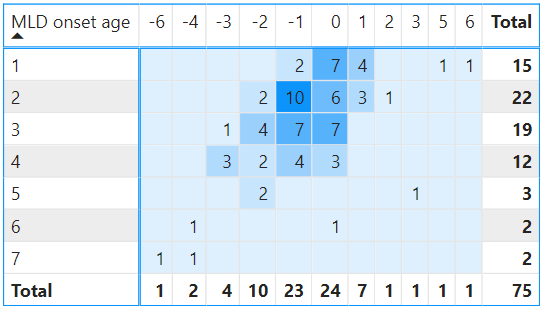 |

II.

| 1. Specific developmental disorder of motor function (F82) | 1. Muscle weakness, generalized (M62.81) |
| --- | --- |
| 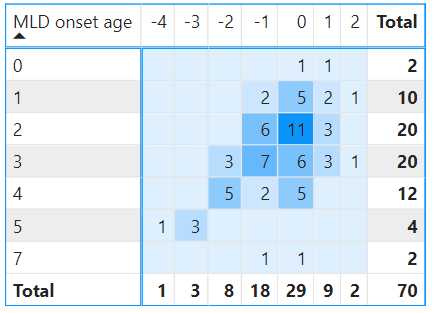 | 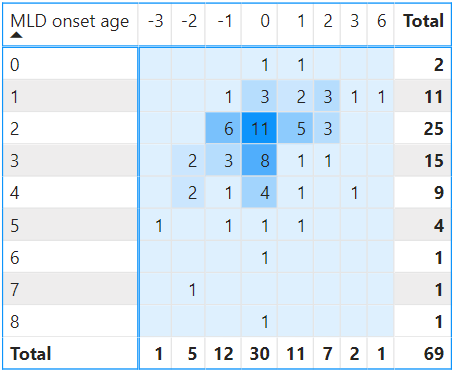 |
| 1. Cramp and spasm (R25.2) | 1. Dysphagia, oral pharyngeal phase (R13.12) |
| 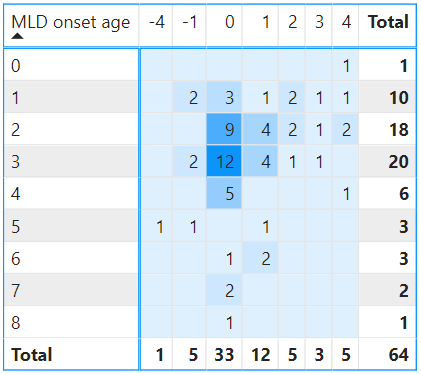 | 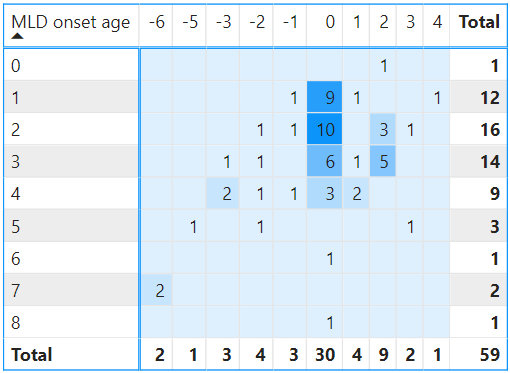 |
| 1. Other specific disorder of muscle (M62.89) | 1. Other symptoms and signs involving the musculoskeletal system (R29.898) |
| 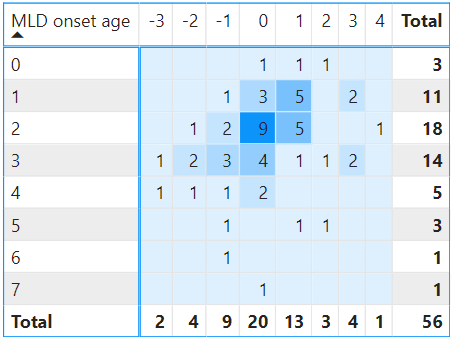 | 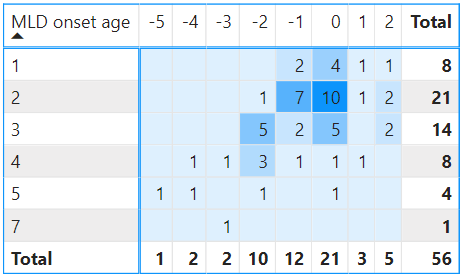 |

III.

| 1. Other abnormalities of gait and mobility (R26.89) | 1. Mixed receptive-expressive language disorder (F80.2) |
| --- | --- |
| 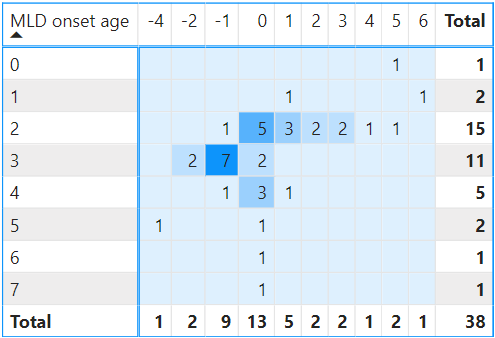 | 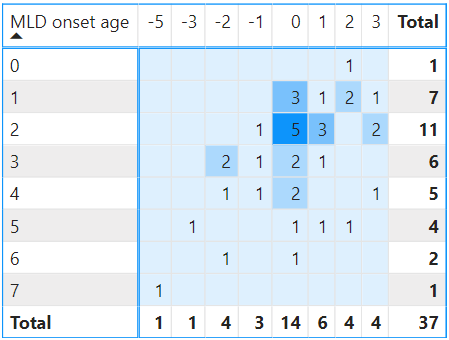 |
| 1. Weakness (R53.1) | 1. Dystonia, unspecified (G24.9) |
| 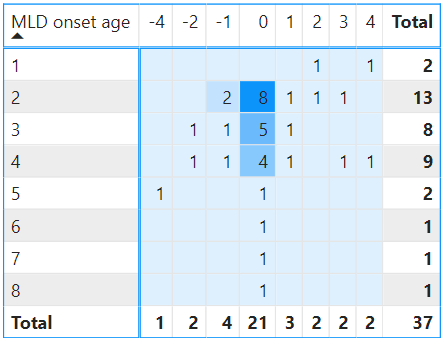 | 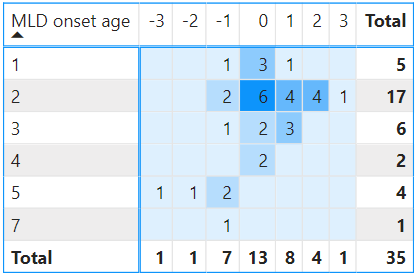 |
| 1. Unspecified abnormalities of gait and mobility (R26.9) | 1. Altered mental status, unspecified (R41.82) |
| 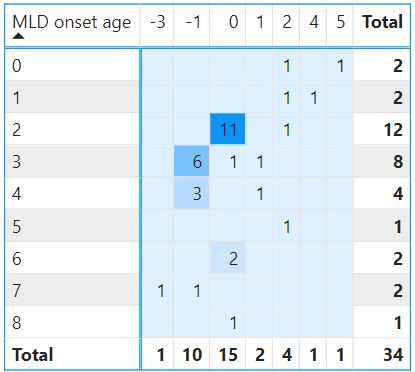 | 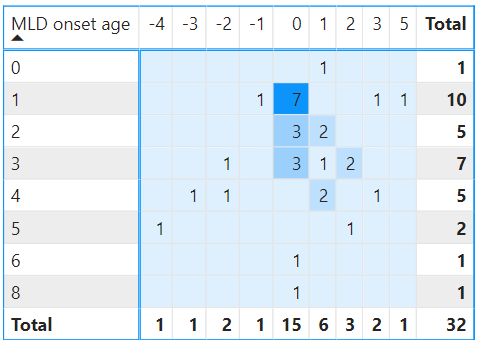 |

**Supplemental Figure 3.** **Signs and symptoms prior to diagnosis in MLD by medical coding within a payor database by sex.** All events coded in the medical system prior to diagnosis of MLD were captured. Events were clustered into clinical feature: suspicion of neurologic disease (n=95), seizure (n=48), language (n=41), neuro (all other; n=138), gastrointestinal or feeding concerns (n=137), and ophthalmologic (n=46). The median time to diagnosis is shown by the symbol with the interquartile range shown in the error bars. Female cohort is shown by dark circle; male cohort by light triangle.

**Supplemental Table 1.** **Primary claims cohort time to diagnosis by sex.** Multiple statistical approaches were used to compare time from first symptom cluster to diagnosis by sex, including Cox Proportional Hazards (logrank; p < 0.05: significant difference in the instantaneous risk of E75.25 diagnosis); Kaplan Meier (KM) survival (logrank; p < 0.05: significant difference in the time-dependent probability of acquiring an E75.25 diagnosis); Schoen: test on time-dependence of scaled Schoenfeld residuals (p > 0.05: no significant evidence of violation of assumption regarding constant proportional hazard over time), and Wilcoxon (W) rank sum (aka Mann-Whitney U) test (p <0.05: significant difference in distribution of time to E75.25 diagnosis (non-parametric test to compare medians). Abbreviations: F: female; M: male; TTD: time to MLD diagnosis (days); Cox: Cox Proportional Hazards (logrank); Kaplan Meier survival (logrank); Schoen: test on time-dependence of scaled Schoenfeld residuals; W: Wilcoxon rank sum (aka Mann-Whitney U) test.

| Measure  Cluster | Count (N) | | | Median TTD  (IQR) | | | p values | | | |
| --- | --- | --- | --- | --- | --- | --- | --- | --- | --- | --- |
|  | **Total** | **F** | **M** | **All** | **F** | **M** | **Cox** | **KM** | **Schoen** | **W** |
| Neurologic features | 138 | 69 | 69 | 256.5  (90.25 – 502.25) | 246.0  (64 – 422) | 287  (109 – 575) | 0.1812 | 0.1792 | 0.7375 | 0.1826 |
| Gastrointestinal/Feeding | 137 | 66 | 71 | 231  (82 – 572) | 205.5  (42.5 – 586) | 231  (102.5 – 564) | 0.2373 | 0.2371 | 0.9482 | 0.2893 |
| Language/Cognition | 41 | 21 | 20 | 267  (100 – 572) | 340  (118 – 546) | 191  (98.25 – 582) | 0.7208 | 0.7089 | 0.5461 | 0.5397 |
| Ophthalmology | 46 | 19 | 27 | 362  (63.5 – 574.5) | 258  (55 – 575.5) | 374  (77.5 – 563) | 0.7479 | 0.7484 | 0.6045 | 0.6079 |
| Seizures | 48 | 23 | 25 | 235.5  (83.25 – 746.75) | 265  (44 – 571.5) | 196  (102 – 876) | 0.1180 | 0.1165 | 0.3473 | 0.3268 |
| Suspicion of neurologic disease | 95 | 42 | 53 | 143  (40 – 444.5) | 161  (21 – 485) | 129  (48 – 395) | 0.7897 | 0.7898 | 0.7431 | 0.6995 |

**Supplemental Table 2.** The Symphony database captured the key medical events prior to diagnosis in a complementary claims dataset.

| Patient count (N) | Dx code | Description |
| --- | --- | --- |
| 152 | R62.50 | UNSPECIFIED LACK OF EXPECTED NORMAL PHYSIOLOGICAL DEVELOPMENT IN CHILDHOOD |
| 124 | R63.3 | FEEDING DIFFICULTIES |
| 104 | R13.10 | DYSPHAGIA, UNSPECIFIED |
| 98 | R62.51 | FAILURE TO THRIVE (CHILD) |
| 83 | F88 | OTHER DISORDERS OF PSYCHOLOGICAL DEVELOPMENT |
| 75 | R62.0 | DELAYED MILESTONE IN CHILDHOOD |
| 70 | F82 | SPECIFIC DEVELOPMENTAL DISORDER OF MOTOR FUNCTION |
| 69 | M62.81 | MUSCLE WEAKNESS (GENERALIZED) |
| 64 | R25.2 | CRAMP AND SPASM |
| 59 | R13.12 | DYSPHAGIA, OROPHARYNGEAL PHASE |
| 56 | M62.89 | OTHER SPECIFIED DISORDERS OF MUSCLE |
| 56 | R29.898 | OTHER SYMPTOMS AND SIGNS INVOLVING THE MUSCULOSKELETAL SYSTEM |
| 38 | R26.89 | OTHER ABNORMALITIES OF GAIT AND MOBILITY |
| 37 | F80.2 | MIXED RECEPTIVE-EXPRESSIVE LANGUAGE DISORDER |
| 37 | R53.1 | WEAKNESS |
| 35 | G24.9 | DYSTONIA, UNSPECIFIED |
| 34 | R26.9 | UNSPECIFIED ABNORMALITIES OF GAIT AND MOBILITY |
| 32 | R41.82 | ALTERED MENTAL STATUS, UNSPECIFIED |
| 31 | F80.9 | DEVELOPMENTAL DISORDER OF SPEECH AND LANGUAGE, UNSPECIFIED |
